# An MPS-Based 50plex Microhaplotype Assay for Forensic DNA Analysis

**DOI:** 10.3390/genes14040865

**Published:** 2023-04-04

**Authors:** Ranran Zhang, Jiaming Xue, Mengyu Tan, Dezhi Chen, Yuanyuan Xiao, Guihong Liu, Yazi Zheng, Qiushuo Wu, Miao Liao, Meili Lv, Shengqiu Qu, Weibo Liang

**Affiliations:** 1 Department of Forensic Genetics, West China School of Basic Medical Sciences and Forensic Medicine, Sichuan University, No. 17, Section 3, Renmin South Road, Wuhou District, Chengdu 610041, China; zhangranranagnes@163.com (R.Z.); xuejohn55@gmail.com (J.X.);; 2 West China Forensics Center, Sichuan University, No. 16, Section 3, Renmin South Road, Wuhou District, Chengdu 610041, China; 3 Department of Immunology, West China School of Basic Medical Sciences and Forensic Medicine, Sichuan University, No. 17, Section 3, Renmin South Road, Wuhou District, Chengdu 610041, China

**Keywords:** microhaplotype, massively parallel sequencing (MPS), Southwest Chinese Han, forensic DNA analysis, degraded mixtures, noninvasive prenatal paternity testing (NIPPT)

## Abstract

Microhaplotypes (MHs) are widely accepted as powerful markers in forensic studies. They have the advantage of both short tandem repeats (STRs) and single nucleotide polymorphisms (SNPs), with no stutter and amplification bias, short fragments and amplicons, low mutation and recombination rates, and high polymorphisms. In this study, we constructed a panel of 50 MHs that are distributed on 21 chromosomes and analyzed them using the Multiseq multiple polymerase chain reaction (multi-PCR) targeted capture sequencing protocol based on the massively parallel sequencing (MPS) platform. The sizes of markers and amplicons ranged between 11–81 bp and 123–198 bp, respectively. The sensitivity was 0.25 ng, and the calling results were consistent with Sanger sequencing and the Integrative Genomics Viewer (IGV). It showed measurable polymorphism among sequenced 137 Southwest Chinese Han individuals. No significant deviations in the Hardy–Weinberg equilibrium (HWE) and linkage disequilibrium (LD) were found at all MHs after Bonferroni correction. Furthermore, the specificity was 1:40 for simulated two-person mixtures, and the detection rates of highly degraded single samples and mixtures were 100% and 93–100%, respectively. Moreover, animal DNA testing was incomplete and low depth. Overall, our MPS-based 50-plex MH panel is a powerful forensic tool that provides a strong supplement and enhancement for some existing panels.

## 1. Introduction

Microhaplotypes (MHs) are novel genetic markers, proposed by the Kidd lab in 2013, to complement current DNA genotyping tools used in forensic genetics [1,2]. They are characterized by the presence of two or more closely linked single nucleotide polymorphisms (SNPs) within 300 bp, with three or more alleles (haplotypes). Therefore, they provide more information than single SNPs, and exhibit a low rate of recombination over such short distances (assuming an average of 1% recombination per megabase and no recombination hotspots within the locus) [3,4]. Microhaplotypes do not preferentially amplify certain alleles within a locus because all alleles at a locus are the same size. Compared to short tandem repeats (STRs), MHs have no stutters, lower mutation rates, and fewer alleles [1]. A large set of MHs can approach the same discrimination power as a set of STRs [5] and provide valuable information on individual identification, mixture interpretation, ancestry prediction, kinship testing, and medical diagnostic applications [1,6,7]. Therefore, they are gaining popularity in the forensic DNA field and have been applied in different related studies [7,8,9,10,11,12].

Currently, massively parallel sequencing (MPS) is the mainstream method for detecting MHs [7]. Sanger sequencing was the “gold standard” method for DNA sequencing [13]. However, when two or more loci are heterozygous, Sanger sequencing cannot determine the cis-trans relationship between alleles of a single SNP in genomic DNA [6,14], i.e., the haplotype phase [15]. Our previous research showed that although the capillary electrophoresis (CE) platform can phase MHs, it only resolved those composed of two SNPs, with a low detection throughput at one time [16,17]. However, MPS can compensate for the deficiencies in Sanger sequencing and CE platforms. It can identify every parental MH allele at a specific locus by clonal amplification, followed by sequencing every amplicon of every DNA strand present in the sample, regardless of its origin from a single or mixed source [1,7,18]. In addition, MPS provides a high sequencing throughput and can simultaneously detect hundreds of thousands of variations. Thus, it enables the forensic analysis of MHs defined by multiple SNPs, and the combination of different SNP alleles within a single short locus can provide a greater probability of individual identification [5,9,11,19,20,21,22]. Thus, MPS technology, which enables clonal sequencing of paternal haplotypes on paternal and maternal chromosomes, has greatly enhanced the characterization of forensic MHs.

Internationally reported panels have successfully developed different sets of MHs [1,2,6]. Thus, an increasing number of identity-, ancestry-, and mixture-informative MHs have recently been published and made available to the global forensic community [5,9,19,20,22,23]. The analysis of these markers and population genetic data will serve as the basis for the future implementation of MH DNA analysis in casework. When a person of interest (POI) cannot be excluded as a possible donor of forensic biological evidence, population-specific allele frequencies are used to estimate the statistical weight of the evidence. Similar to traditional STRs, the application of MH sequencing in casework requires the development of large and appropriate allele frequency (AF) datasets [24]. Nonetheless, Kidd et al. have collected the AF data of initial MHs among the global population and uploaded it to the ALFRED (ALelle FREquency Database) [25]. However, ALFRED does not include the MH AF of the Southwest Chinese Han population (Chengdu City), which would hinder relevant forensic application research. Although the MicroHapDB (Microhaplotype Database) established by Standage et al. [26] includes the basic parameters of 412 MHs in 26 populations, it only includes the published MHs. These markers were selected from the original MH pools by different researchers for certain purposes or in specific populations. However, t most of the works do not release the data of the original MH pools, which limits the marker selection of other researchers to those published MHs. It may be difficult to meet other different research needs sometimes [27].

To fill this gap, in this study, we extracted the “original loci pool of MHs” of the Chinese Southern Han (CHS) from the 1000 Genomes Project (Phase 3) using our developed MHs screening software combined with the PHASE software. Thus, after a series of extractions and optimizations, we constructed 50 MHs (251 SNPs) on 21 autosomes using a MultipSeq^®^ multiple polymerase chain reaction (multi-PCR) targeted capture sequencing protocol based on MPS. From this, we developed an MPS-based 50-plex MH panel to obtain the genotypes of 137 Southwest Chinese Han individuals, and calculated AF and forensic statistical parameters for each sample. We then characterized the efficiency of custom probe detection based on depth of coverage (DoCs) and allele coverage ratios (ACRs). Moreover, we demonstrated the applicability of the protocol by analyzing the sensitivity, accuracy, specificity, population genetics, simulated degraded samples, simulated mixtures, and real animal samples. Compared to commonly used autosomal STRs [28], SNPs [29], or published MH panels [16,30,31,32], the results showed that our 50plex MH panel provided higher genetic polymorphism and held a greater potential for forensic applications, such as individual identification, degradation detection, mixture interpretation, kinship analysis, etc.

## 2. Materials and Methods

### 2.1. MH Selection

We used the homemade MH screening software combined with PHASE v2.1.1 (https://stephenslab.uchicago.edu/phase/download.html, accessed on 1 January 2022, Seattle, WA, USA) to analyze the 1000 G data (Phase 3). Based on a previous study by our research group [27], we extracted MHs consisting of two or more SNPs within 80 bp in the CHS and an effective number of alleles (A_e_) value ≥ 3, and estimated the theoretical value of population haplotype frequencies. On this basis, we screened candidate MHs according to the following criteria: (1) all SNPs of MHs must show a minor allele frequency (MAF) > 0 in the dbSNP database; (2) an A_e_ value ≥ 4 because MHs with high A_e_ can enhance individual identification, mixture interpretation, and kinship analysis [18]; (3) the MH with the largest A_e_ from all overlapping sequences in each group, taking each autosome as a unit; (4) the MHs with apparent repeat motifs in the base sequence were removed; (5) the initial set of MHs with a physical position ≥10 Mb were selected as an interval to avoid linkage disequilibrium (LD) among the selected MHs; and (6) only MHs for which functional primers could be designed.

### 2.2. Primer Design

After obtaining the candidate MHs, we handed over the region of interest (ROI), that is, the physical location information of the MHs, to iGeneTech Biotechnology Beijing Co., Ltd. using the online MFEprimer v3.1 (https://mfeprimer3.igenetech.com/muld, accessed on 19 January 2022, Beijing, China) to design and validate multiple PCR primers that targeted the genomic sequence of the MHs in our panel. Based on thermodynamic stability [33], highly specific multiplex primers were designed on both sides of the ROI; the amplicon was 120–200 bp. We then evaluated primer dimerization and non-specific amplification, tested the designed and synthesized primers, and replaced primers with a poor detection effect.

### 2.3. Sample Collection

Peripheral blood samples of 137 unrelated Southwest Chinese Han individuals were collected after obtaining informed consent with the approval of the Medical Ethics Committee of Sichuan University (No. KS2022770). Genomic DNA over 18 ng/μL, extracted using the phenol-chloroform method, were quantified using the Qubit™ dsDNA HS Assay Kit on a Qubit^®^ 4.0 Fluorometer according to the manufacturer’s protocol (https://assets.thermofisher.com/TFS-Assets/LSG/manuals/MAN0017209_Qubit_4_Fluorometer_UG.pdf, accessed on 14 April 2022, Thermo Fisher Scientific, Waltham, MC, USA).

### 2.4. Sensitivity Design and Accuracy Verification

For the sensitivity study, 10, 5, 1, 0.5, 0.25, and 0.125 ng of 2800 M control DNA (Promega, Madison, WI, USA) were input into the MPS platform. All DNA libraries were prepared manually and run on an Illumina^®^ NovaSeq^TM^ 6000 system, according to the manufacturer’s protocol (https://emea.support.illumina.com/downloads/novaseq-6000-system-guide-1000000019358.html, accessed on 9 October 2022, Illumina, San Diego, CA, USA). Eighteen samples (1 sample × 6 gradients × 3 replicates) were placed on the same NovaSeq 6000 chip.

Seven unrelated samples were randomly selected, and their original bam files obtained from MPS were input into the Integrative Genomics Viewer (IGV) v2.16.0 (https://software.broadinstitute.org/software/igv/userguide, accessed on 10 October 2022, Cambridge, MA, USA) to analyze the genotype of all the target 50 MHs. Among them, two MH loci and four unrelated samples were randomly selected for Sanger sequencing (Tsingke Biotechnology Co., Ltd., Beijing, China). Finally, the MH genotypes, obtained using the pipelines developed by our laboratory, were compared with those obtained by IGV and Sanger sequencing simultaneously.

### 2.5. Library Preparation and Sequencing

**Figure 1:**
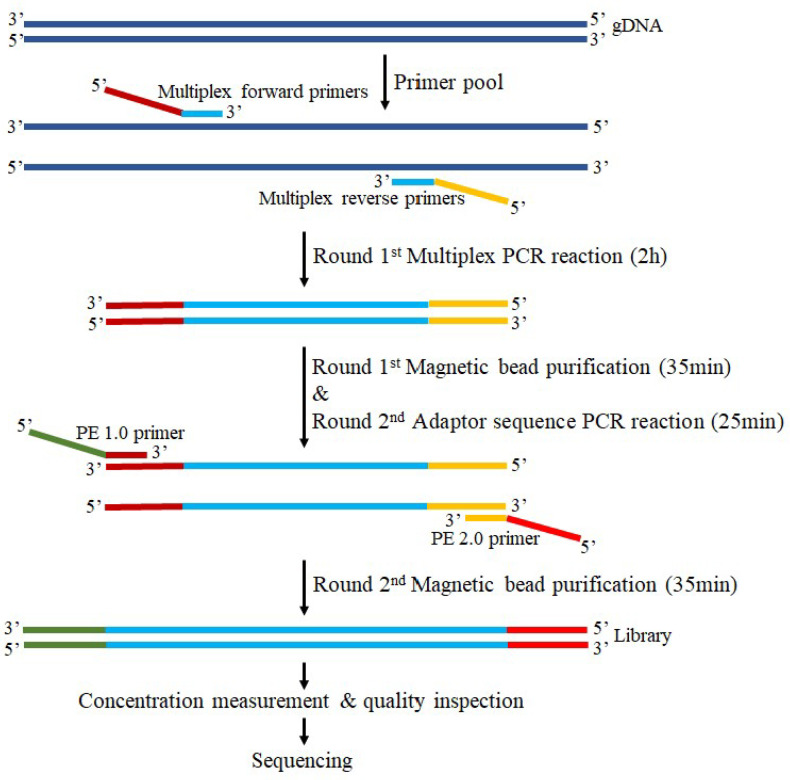
Library construction and capture workflow of the multiple polymerase chain reaction (multi-PCR). All samples followed the standard sequencing workflow with a total time course of 3 h 35 min.

Library preparation and multiplex capture for ROI sequencing were performed following the procedure shown in Figure 1, according to the manufacturer’s protocol (see Section 2.4).

The first round of multiple PCR reactions is to obtain the amplicon product of the target region. By the NovaSeq 6000 S4 Reagent Kit v1.5, the multiple PCR reaction system contained 3.5 μL of Enhancer buffer NB (1N), 2.5 μL of enhancer buffer M, 10 μL of IGT-EM808 polymerase, 5 μL of primer pool, 1–5 ng a DNA/reaction tube, and finally made up to 30 μL with ddH_2_O. The multiple PCR reaction conditions consisted of a preincubation at 95 °C for 3 min 30 s, followed by 22 cycles of 98 °C for 20 s, 60 °C for 4 min, and a final extension at 72 °C for 5 min on an ETC811 PCR thermocycler (Dongsheng Innovation Biotechnology Co., Ltd., Beijing, China) using a customized MultipSeq^®^ Custom Panel (iGeneTech Biotechnology Beijing Co., Ltd., Beijing, China) with amplicons between 120 and 200 bp. The pure amplification product was obtained through the first round of magnetic bead purification, which was used as the template for the second round of PCR reaction.

In the second round of adapter PCR reaction, sequencing adapters were introduced to both sides of the amplicon product to obtain a library. The adapter sequence PCR reaction system contained 2.5 μL of Enhancer buffer M, 10 μL of IGT-EM808 polymerase, 2 μL of CDI Primer (premix adapter primer), 13.5 μL of PCR product mixture, and finally made up to 30 μL with ddH_2_O. The adapter sequence PCR reaction conditions consisted of a preincubation at 95 °C for 3 min 30 s, followed by 9 cycles of 98 °C for 20 s, 58 °C for 60 s, 72 °C for 30 s, and a final extension at 72 °C for 5 min on an ETC811 PCR thermocycler. The pure amplicon library was obtained through the second round of magnetic bead purification.

The obtained library was then subjected to strict concentration measurements using the Qubit™ dsDNA HS Assay kit and the Qsep400™ system for quality inspection according to the manufacturer’s protocol (https://apps.bioptic.com.tw/webdl/Instrument/F0043_Qsep400%20Operation%20Manual-%20Hardware%20-ENG-E.pdf, accessed on 9 October 2022, BiOptic, New Taipei City, Taiwan, China). Subsequently, sequencing was performed on an Illumin^®^ NovaSeq^TM^ 6000 system using amplicon-targeted capture in PE150 paired-end sequencing mode.

### 2.6. Sequencing Data Analysis

The raw image data obtained after sequencing were converted and deduplicated from base calling files using the bcl2fastq v2.20.0.422 (Illumina, San Diego, CA, USA). The resulting raw sequencing sequences (FASTQ files) were submitted to Trimmomatic v0.38 (Max Planck Institute, Potsdam, BB, Germany) and FastQC v0.11.3 (Babraham Institute, Cambridge, UK) in-house quality control software to remove low-quality reads, followed by the Bwa v0.7.12 (Wellcome Trust Sanger Institute, Cambridge, UK) [34] and Samtools to align them with the reference human genome (Hg19, GRCh37). Single BAM files were submitted to variant calling at SNP/INDEL sites using Samtools v1.9 (UChicago, Chicago, IL, USA) and Varscan v2.4.3 (UWashington, Seattle, WA, USA) to generate VCF files [35]. Raw identification calls for SNV and InDels were further filtered using the thresholds read depth > 4, mapping quality > 20, and variant quality score > 20. Variation loci were annotated using Annovar v201707 (UPenn, Philadelphia, PA, USA). Annotation databases included ExAC, ESP6500, 1000 Genomes, gnomAD, SIFT, CADD, and Polyphen 2. We then used our laboratory pipelines for MH calling using the CIGAR and MD: Z tag information of BAM files [12]. The minimum DOC for each target region and threshold for each MH allele were set to 100× and 25×, respectively, for further analysis. After initial filtering with a threshold of 25 reads, the default minimum read coverage for an allele was set at 5%. If the number of reads for an allele are below this value, the alleles will not be called. The default minimum value for allele frequency for heterozygous markers was set at 10%. If two or more alleles are detected at a marker, any single allele must have coverage of at least this percentage of total reads at the marker to be called. The default minimum value for allele frequency for homozygous markers was set at 90%. A single allele at a marker must have coverage of at least this percentage of total reads at the marker to be called.

We displayed the alleles of each MH and compiled the DoCs (i.e., depth of sequencing) and ACRs in an Excel output format. The ACR was defined as the lower coverage of the allele at a heterozygous locus divided by the higher coverage in a single gDNA sample. It is commonly used to assess the balance between the two alleles of heterozygotes detected by high-throughput sequencing of genetic markers.

### 2.7. Statistical Analysis

Based on the above pipeline, we obtained the allelic genotype, AF, and forensic statistical parameters of 50 MHs among 137 Southwest Chinese Han individuals, including homozygosity (Hom), heterozygosity (Het), match probability (MP), discrimination power (DP), probability of exclusion (PE), polymorphism information content (PIC), and the typical paternity index (TPI) by using the Modified-Powerstates v. 1.2 (Promega, Madison, WI, USA) [36]. Then we used the following formula to calculate combined match probability (CMP), combined discrimination power (CDP), and combined probability of exclusion (CPE), respectively, including CMP = 1 − ΣP(1 − MP_1_) (1 − MP_2_) (1 − MP_3_) … (1 − MP_50_), CDP = 1 − ΣP(1 − DP_1_) (1 − DP_2_) (1 − DP_3_) … (1 − DP_50_) and CPE = 1 − ΣP(1 − PE_1_) (1 − PE_2_) (1 − PE_3_) … (1 − PE_50_), where 1 … 50 represent the 50 MHs. The A_e_ value was calculated as the reciprocal of homozygosity: 1/∑p_i_^2^, where p_i_ is the frequency of allele i and summation includes all alleles at the MH. In addition, the Hardy–Weinberg equilibrium (HWE) *p*-value and LD value were calculated using Arlequin v3.5 (University of Berne, Lausanne, Switzerland) [18,37].

### 2.8. Mixture Design

Two unrelated individuals were randomly selected to simulate the two-person DNA mixtures. The minor DNA amount was fixed at 0.5 ng, and different major DNA amounts were then added to form mixtures at ratios of 1:1, 1:3, 1:5, 1:10, 1:20, and 1:40. For MPS detection to evaluate the efficiency of the panel, 1 μL of each mixture was used. All mixtures were prepared using TE (Solarbio Science & Technology Co., Ltd. Beijing, China) and sterile 0.2 mL amplification tubes (Axygen Scientific, Union City, CA, USA), and samples were stored at −20 °C until use. The degree of mixing was detected using the AGCU EX22 kit (Applied ScienTech, Suzhou, Jiangsu, China) on an ABI 3500 Genetic Analyzer according to the manufacturer’s protocol (https://tools.thermofisher.com/content/sfs/manuals/4401661.pdf, accessed on 14 July 2022, Applied Biosystems, Thermo Fisher Scientific, Waltham, MC, USA). The results were analyzed using the GeneMapper ID-X v1.2 according to the manufacturer’s protocol (https://assets.thermofisher.com/TFS-Assets/LSG/manuals/cms_072557.pdf, accessed on 17 July 2022, Applied Biosystems, Thermo Fisher Scientific, Waltham, MC, USA).

### 2.9. Degradation Design

To simulate single-source degraded samples, two randomly extracted DNA samples were diluted to a concentration of 5 ng/μL and treated with DNase I (Thermo Fisher Scientific, Waltham, MC, USA), respectively [38]. Subsequently, 45 μL of intact DNA (5 ng/μL) was mixed with 3.75 μL of 10× MgCl_2_ buffer (Thermo Fisher Scientific, Waltham, MC, USA). To the mixture, 0.6 μL of 0.3 U/μL DNase I was added, followed by incubation at 37 °C, after which 10 μL of degraded DNA from the incubated mixture was removed at predetermined time intervals (2.5, 5, 10, and 15 min, respectively), and placed in separate sterile 0.2 mL amplification tubes (Axygen Scientific, Union City, CA, USA), respectively. EDTA (1.6 μL, 30 mM) was immediately added to each tube and incubated at 65 °C for 10 min to stop DNA degradation. The degree of degradation was then evaluated using the AGCU EX22 Kit on an ABI 3500 Genetic Analyzer and the High Sensitivity DNA Kit on an Agilent 2100 Bioanalyzer according to the manufacturer’s protocol (https://www.agilent.com/cs/library/usermanuals/public/2100_Bioanalyzer_Expert_USR.pdf, accessed on 30 July 2022, Agilent Technologies, Santa Clara, CA, USA). For the MPS, 1 μL of each sample treated with DNase I was used.

To simulate mixed degradations, one of the above single-source degradations was set as the minor DNA and fixed at 0.5 ng, and the other was set as the major DNA. The major DNA, degraded at different times, was added to corresponding minor DNA to form mixtures at a ratio of 1:10. The subsequent evaluation and detection processes of degraded degrees were the same as the above single-source degradations. For the MPS, 1 μL of each 1:10 mixed degradation was used.

### 2.10. Species Specificity

We tested common animal DNA to assess the specificity of our panel because non-human DNA may be present in forensic biological evidence. Thus, animal DNA samples from cats, bovines, chickens, ducks, fish, pigs, rabbits, and sheep were sequenced using multi-PCR targeted capture sequencing in the same manner as human DNA, with an input DNA amount of 3.753–6.506 ng.

## 3. Results

### 3.1. MH Selection and Primer Design

**Figure 2:**
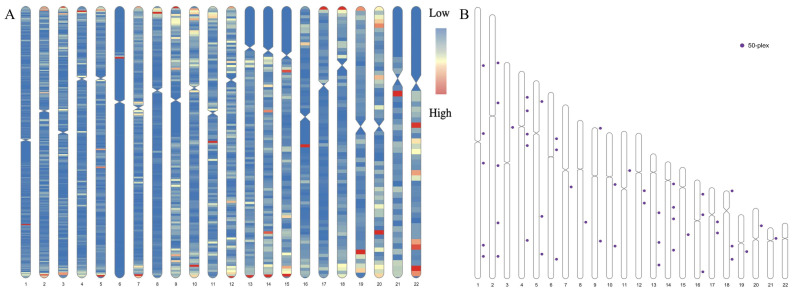
Distribution of target microhaplotypes (MHs). (**A**) Distribution of MHs within 80 bp consisting of 2 or more single nucleotide polymorphisms (SNPs) in the Chinese Southern Han (CHS) and effective number of alleles (A_e_) values ≥ 3. The “low” and “high” represent the distribution densities of the target MHs. (**B**) Physical locations of the final 50 MHs.

A total of 178 candidate MHs were screened from 1000 G (Phase 3), and the MPS-based protocol allowed primer design and multiplex detection of 128 of these MHs in a single assay. Six rounds of optimization were performed on the initially constructed panel using six samples (the company’s internal standard DNA H01, 2800 M, and four experimental samples). Some MHs were excluded, such as those with many nonspecific amplification products, large amplification and sequencing deviations between different samples, and low sequencing coverage. Fifty MHs were reserved to ensure the best system performance of the panel (Table 1, Figure 2) and distributed on 21 autosomes (no target MH on chr22 after six rounds of optimization). We observed 1–5 MHs on each autosome (average 2.38), with each MH comprising 3–15 SNPs (total 251, average 4.83), marker lengths of 11–81 bp (average 65.58 bp), and an amplicon of 123–198 bp (average 156.02 bp). Specific information on the 50 MHs and primers is provided in Supplementary Table S1.

### 3.2. Sensitivity and Accuracy Analysis

For three replicates with different inputs of 2800 M (10, 5, 1, 0.5, 0.25, and 0.125 ng), we detected complete profiles for all 50 MHs at 0.25 ng. Only one MH (MH-37) dropout was observed in the third replicate at 0.125 ng, as the reads were 20×, which is below the analytical threshold of 25× (Supplementary Figure S1A). The overall DoCs were 801.24–11,010.84× (average 5623.39×) and decreased gradually with decreasing DNA input (linear correlation coefficient R^2^ = 0.8814) (Supplementary Figure S1B). The minor DNA of the non-degraded and degraded mixtures in the next simulation study was fixed at 0.5 ng.

**Figure 3:**
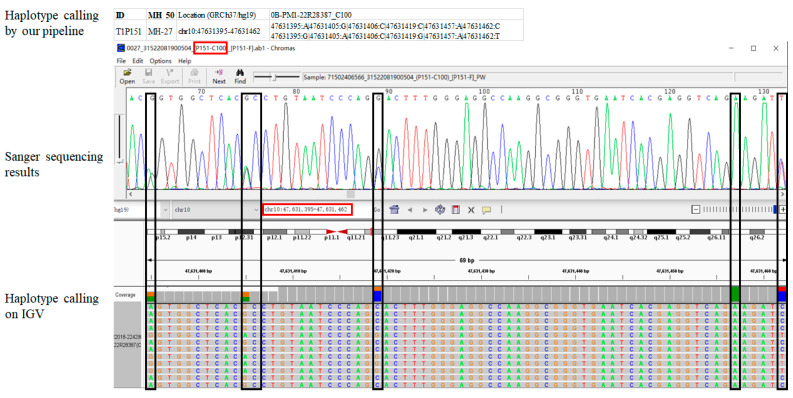
An example of three analysis methods for MH-27 in sample C100. The entire figure shows the genotypes obtained by our pipeline, Sanger sequencing, and Integrative Genomics Viewer (IGV) from top to bottom. The black boxes indicate the target SNPs. The two red boxes represent “MH ID-Sample ID” and “Location (GRCh37/hg19)”, respectively. The screenshot only displays the physical location and length of the target MH.

The MH, sample numbers, and Sanger primers are shown in Supplementary Table S2. We did not observe inconsistent haplotypes among Sanger sequencing, IGV, or our pipeline in the analyzed MH loci or unrelated individuals. Figure 3 shows the corresponding genotypes of the three analysis methods for a random MH in a random sample. The results showed 100% concordance. Supplementary Figure S2 presents the remaining examples.

### 3.3. Panel Performance

**Figure 4:**
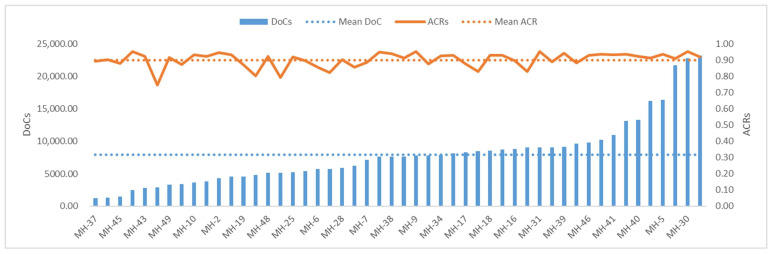
The depths of coverage (DoCs) and allele coverage ratios (ACRs) of 50 MHs based on 137 sequenced Southwest Chinese Han unrelated individuals. The blue bars on the left axis represent the overall DoCs in ascending order; the dashed blue line represents the mean DoC; the solid orange line on the right axis represents the overall ACRs; and the dashed orange line represents the mean ACR.

Fifty MHs of all 137 unrelated Southwest Chinese Han individuals in this study were consistently captured and sequenced to obtain complete MH alleles. These samples were genotyped at 1.825–25.992 ng of input DNA using the DoCs and ACRs of all 50 MHs to assess the panel sequencing performance. The average DoC was 7928.39 ± 4990.952× (Figure 4). The average ACR was 0.90 ± 0.045, and 96% of the MHs (48/50) exhibited a proportion of allele balance ≥ 80% (Figure 4), indicating the panel had a good balance in detecting heterozygotes (i.e., good heterozygosity balance). No correlation was found between the DoCs and ACRs (linear correlation coefficient R^2^ = 0.0771).

### 3.4. Polymorphism Information

**Figure 5:**
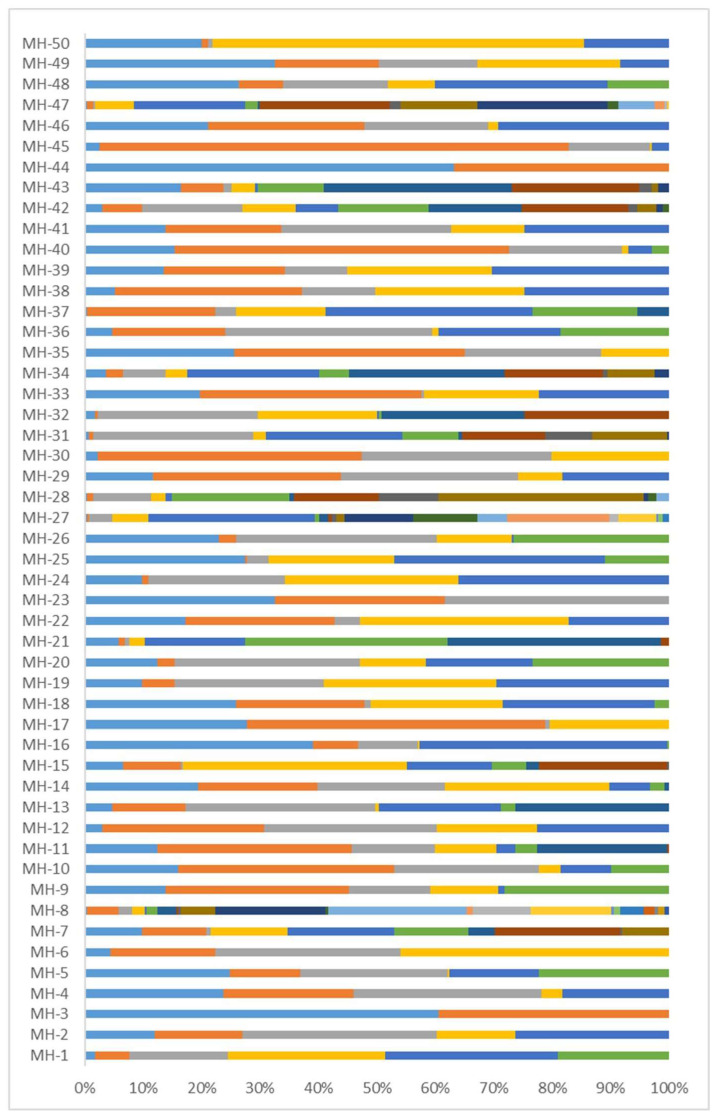
Allele frequencies of 50 MHs based on sequenced 137 Southwest Chinese Han unrelated individuals. Different colors of the bars represent different haplotypes (alleles), and the length of each bar represents the frequency of each allele.

All the 50 MHs in our panel were successfully sequenced. Haplotype (i.e., allele) frequencies calculated from sequencing data from all 137 unrelated individuals are shown in Figure 5 and Supplementary Table S3. Each MH had 2–23 alleles (average 7), of which 3 MHs showed 2–3 alleles, 4 MHs showed 4 alleles, 15 MHs showed 5 alleles, 12 MHs showed 6 alleles, 3 MHs showed 7 alleles, and 13 MHs showed 8 or more alleles. The frequencies of all the 350 alleles ranged from 0.004–0.803.

**Figure 6:**
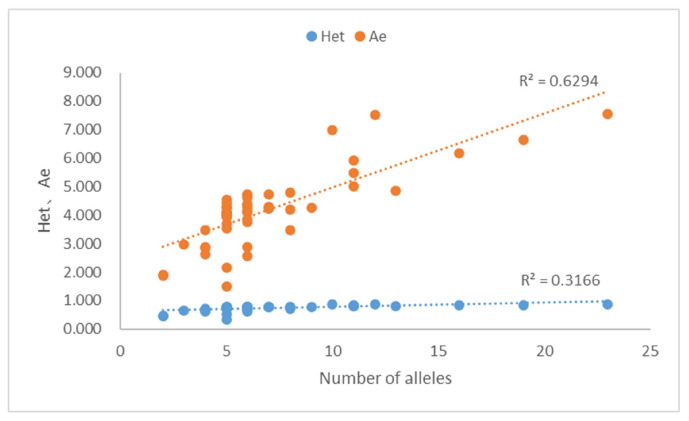
Scatter plot of 50 MHs for the number of alleles, heterozygosity (Het) and A_e_. Relationship among the number of alleles, Het, and A_e_ values.

Based on allele frequencies (Supplementary Table S3), forensic parameters (Supplementary Table S4) showed that the Hom, Het, and A_e_ were 0.133–0.665 (average 0.266), 0.335–0.867 (average 0.734), and 1.503–7.547 (average 4.192), respectively. Among the 50 MHs, 10 A_e_ were <3.0, 8 A_e_ were ≥3.0, 24 A_e_ were ≥4.0, 3 A_e_ were ≥5.0, 3 A_e_ were ≥6.0, and 2 A_e_ were ≥7.0. We observed that both the A_e_ and Het increased with increasing alleles, with R^2^ of 0.6294 and 0.3166, respectively (Figure 6). Meanwhile, A_e_ increased with increasing Het (R^2^ = 0.9222, Supplementary Table S4). The highest Het (0.801–0.867) also had the highest A_e_ (5.026–7.547). In general, Het and A_e_ were larger when there were more alleles of an MH, and the frequency of each allele tended to be the same.

We also observed that the MP, CMP, DP, CDP, PE, CPE, PIC, and TPI were 0.032–0.484 (average 0.127), 0.999180791, 0.516–0.968 (average 0.873), 1–3.109 × 10^−49^, 0.086–0.747 (average 0.481), 1–8.727 × 10^−16^, 0.308–0.855 (average 0.692), and 0.770–4.029 (average 2.018), respectively (Supplementary Table S4). Among the 50 MHs, MH-8 showed the highest polymorphism. For MH-8, the Het, A_e_, MP, DP, and PIC were 0.867, 7.547, 0.032, 0.968, and 0.855, respectively. After Bonferroni correction, we observed that all 50 MHs had no significant bias in HWE (*p* = 0.05/50 = 0.001) or LD detection (*p* = 0.05/2485 = 0.00004081) (Supplementary Tables S5 and S6).

### 3.5. Mixture Analysis

The 50-MH panel was developed as a stand-alone forensic panel but could also be used as a complement to STR markers. To explore the detection threshold of the mixture ratio, the simulated two-person mixtures were genotyped after a series of dilutions (1:1, 1:3, 1:5, 1:10, 1:20, and 1:40). Based on the sensitivity results, the minor DNA was fixed at 0.5 ng, and the major DNA was added at the mixing ratio. The AGCU EX22 Kit (Applied ScienTech, Jiangsu, China) can only detect the complete genotype of the major and minor DNA at a 1:1 ratio. Thus, minor DNA was incompletely genotyped at the mixing ratios of 1:3, 1:5, and 1:10, and the identity-informative alleles of STR were partially dropped. Minor DNA was undetectable at 1:20 and 1:40, and the identity-informative alleles of STR completely dropped out (Supplementary Figure S3). The overall DoCs of the MPS-based 50plex MH panel were 24,597.48–41,927.99× (average 31,121.65×) and was able to detect the complete genotype of major and minor DNA at a ratio as low as 1:40, with a maximum number of individual alleles of 132 (Table 2). For a two-person mixture with 1 µL of input DNA, complete MH profiles of the minor DNA were observed at a ratio as low as 1:40, and 100% (61/61) of unique alleles for the minor DNA were reported.

### 3.6. Analysis of Degraded Samples

The lengths of the DNA fragments ranged from 120 to 320 bp after different DNase I treatment times (2.5, 5, 10, and 15 min). The degree of degradation of single and mixed samples detected using the Agilent 2100 Bioanalyzer (Agilent Technologies, Santa Clara, CA, USA) is shown in Supplementary Figure S4. The degradation results were consistent with the fragment distribution of STR genotypes (Supplementary Figure S3). Long-STR genotyping failed when random single DNA was treated with DNase I at 37 °C for 2.5, 5, 10, and 15 min (Supplementary Figure S5A,B). In contrast, the MPS-based 50-MH panel successfully obtained complete alleles in all single degraded DNAs (Supplementary Table S7), with an overall DoCs of 7336.50–18,408.12× (average 14,420.24×). Long STR genotyping failed when the simulated two-person mixtures were treated with DNase I at 37 °C for 2.5, 5, 10, or 15 min (Supplementary Figure S5C). However, the overall DoCs of the MPS-based 50-MH panel were 1464.69–49,182.18× (average 22,211.13×). The complete profiles of the major and minor DNA were successfully obtained in six types of degraded mixtures of 1:10–2.5 and 1:10–5 (except for the poor sequencing result caused by the low-quality library construction of the 1:10–5-1 sample). Only 1–4 unique allele (identity-informative allele) dropouts of minor DNA were observed in the other four degraded mixtures of 1:10–10 and 1:10–15. The overall detection rates were 93–100% (Table 2, Supplementary Table S7). These results suggested that 50plex MHs were more efficient than CE-STRs in sequencing and genotyping degraded single and mixed DNAs.

### 3.7. Species-Specific Analysis

Complete genotypes of 50-plex MHs were not achieved for all eight animal DNA samples with 1 µL of DNA input. For animal DNA, the overall DoCs ranged from 103.00 to 548.00× (average 322.00×) and 2–8 MHs were detected for each DNA (Supplementary Table S8). For MHs, the overall DoCs ranged from 33.00 to 337.00× (average 103.04×), of which only 25 MHs containing 1–4 alleles were genotyped. The current data showed that our panel incompletely genotyped different animal samples with very low signals, so the species specificity of the 50-plex assay is sufficient for routine casework situations.

## 4. Discussion

In this study, we developed a thermodynamic stability-based multiple PCR (i.e., highly specific multiplex primers) capture-sequencing protocol targeting 50 MHs based on the Illumina HiSeq platform. The forensic power of the 50-plex-MH panel in 137 unrelated individuals was evaluated based on DoCs and ACRs. The sensitivity, accuracy, polymorphism, forensic parameters, degraded samples, mixtures, and animal samples of the panel performed adequately, thereby indicating that our panel was a powerful forensic tool and could provide a good supplement and enhancement to existing detection methods. Based on our previous studies of 15 SNP-SNP MHs [16,17], we comprehensively optimized the MH screening, sequencing, and analysis protocols in this study.

Microhaplotypes combine the advantages of STRs and SNPs, with no stutter peak or amplification bias, short markers and amplicons, low mutation and recombination rates, and high polymorphism. They are recognized as powerful markers for various forensic purposes [7,39]. Compared with phased Sanger sequencing and CE platforms, single sequence reads of MPS can cover a wide range of analyzed MHs and are highly informative following MH detection. Therefore, they can be used to analyze true haplotypes. Moreover, MPS is a powerful platform for simultaneously analyzing several target areas and different sample types, thereby addressing relevant forensic questions in a single assay [22].

At present, most MHs of reported panels are selected from published articles [8].Therefore, the current screening method is not systematic, and its genome coverage is not extensive. The number of MHs in some panels is small, and the detection platform still uses first-generation sequencing [16,17,32,40]. Moreover, the analysis methods of some MPS panels, such as Flfinder [11] and MHtyper [41], are more suitable for their own research analyses. These panels are limited by the number of loci, so the performances of polymorphism, forensic parameters, and mixture detection are limited. To compensate for these deficiencies, we aimed to develop a method that quickly and effectively screens short and high-A_e_ MHs sets (including SNPs only) in a target population using our developed MH screening software combined with PHASE software based on the 1000 G [27]. High-throughput sequencing of multiple markers and different sample types was performed using the MPS platform. Finally, automatic sequencing data analysis was performed using our developed pipeline.

We initially selected 178 candidate MHs and retained 50 MHs after six optimizations to ensure the best system efficiency of the panel. Only one of the 50 MHs (MH-32) was included in the Kidd-reported MH panel (mh13KK-218) after comparison with the ALFRED database and other reported MHs. The remaining 49 MHs were novel and unreported (Supplementary Table S1). The marker length and amplicon of the 50 MHs were 11–81 bp (average 65.58 bp) and 123–198 bp (average 156.02 bp), respectively, which were shorter than those of other panels. For example, the marker lengths of 60, 56, 40, 30, and 18 MHs have been reported as 20–116 [42], 17–218 [43], 8–114 [44], 63–423 [30], and 14–103 bp [45], respectively. The amplicons of 74, 56, 30, and 21 MHs have been reported as 157–325 [14], 115–263 [43], 63–423 (average 216) [30], and 125–375 bp [46], respectively.

The Het and A_e_ of the 50 MHs were 0.335–0.867 (average 0.734) and 1.503–7.547 (average 4.192), respectively, which were higher than those of other panels. For example, Oldoni et al. reported that the Het and A_e_ of the 74 MHs were 0.51–0.78 [47] and 1.307–6.010 (median 2.706) [14], respectively. The A_e_ of the 56, 40, and 30 MHs have been reported as 1.74–6.98 (average 3.45) [43], 2.62–4.41 (average 3.61) [44] and 3.91 [30], respectively. Studies have shown that Het > 0.4 and A_e_ > 3.0 loci can be effectively used to analyze individual identification, kinship testing, degradation, mixtures, and ancestral inferences [18]. Therefore, our panel has significant research value for forensic applications. Among the 50 MHs, one MH (MH-24) had three pairs of primers after optimization and testing. The amplicons were the same, and therefore did not affect data analysis.

We added sensitivity gradients of 10, 5, 1, 0.5, 0.25, and 0.125 ng, with three replications showing sensitivities as low as 0.25 ng (Supplementary Figure S1). This provided a theoretical basis for the scientific setting of minor DNA amounts for subsequent studies on non-degraded and degraded mixtures. A multiple PCR-targeted capture and sequencing protocol based on MPS was used to obtain the complete genotypes of 50 MHs from 137 unrelated Southwest Chinese Han individuals. Combined with the sensitivity results, the DNA input for sequencing was 0.25–26 ng; the greater the DNA input, the higher the sequencing depth. The average DoC was 7928.39 ± 4990.95×, the average ACR was 0.90 ± 0.05, and 96% of MHs (48/50) showed an allele balance ratio ≥ 80% (Figure 4), indicating that the sequencing efficiency of our panel was high. Each MH had an average of seven alleles, and 85.7% (300/350) of alleles had a frequency ≥0.01, with the highest being 0.803, indicating good polymorphism in our panel (Figure 5, Supplementary Table S3). The sensitivity of 250 pg is in the range reported for other MPS-based systems used for forensic STR analysis [48] and will be sufficient for many routine applications. For samples with low DNA amounts, such as minute traces, touch DNA, or degraded samples, further improvement of our system will be required.

For sequencing data analysis, we tried the Flfinder we had developed earlier [11], but because of the proximity of SNPs in some MHs, it could not meet the input file format requirements of Flfinder. Therefore, on the basis of Flfinder, we created a set of scripts using the Python and R languages for MHs calling. We compared read thresholds of 15×, 20×, 25×, and 30×, and found that at 25× the alignment accuracy of calling obtained by our pipeline and IGV was the highest, which was also consistent with Sanger sequencing (Figure 3, Supplementary Table S2, Supplementary Figure S2).

Heterozygosity (Het) is the most important parameter for familial identification, as a higher Het at the locus increases the chance that the associated allele will be uncommon in a given population, but is more likely to be found in relatives than in unrelated individuals [3]. In our study, we observed that A_e_ increased with increasing Het, and the highest A_e_ corresponded to the highest Het. This is related to the number and frequency of the alleles in the population. Therefore, the selection of the most informative marker for familial identification depends on the A_e_ value. The A_e_ value is also an important index for evaluating the ability of a mixture analysis [49]. For our 50-plex MH panel, Het values of more than 98% (49/50) of MHs were >0.40, A_e_ values of more than 80% (40/50) MHs > 3.0, and CDP and CPE were 1–3.109 × 10^−49^ and 1–8.727 × 10^−16^, respectively (Supplementary Table S4). The results showed that our panel has surpassed the capacity of commonly used 23 STRs [50,51] or 52 SNPs [52] and several other reported MH panels [30,53], indicating that our panel could be potentially effective for future applications in individual identification, kinship testing, mixture interpretation, and non-invasive prenatal paternity testing (NIPPT) [3,42].

For undegraded mixtures, single-degraded samples, and degraded mixtures, complete STR genotypes could not be detected using the AGCU EX22 Kit (Applied ScienTech, Jiangsu, China) (except for the 1:1 undegraded mixture) (Supplementary Figures S3 and S5). However, our MPS-based panel was able to observe all complete MH genotypes (Table 2 and Table S7). For the degraded mixtures, a ratio of 1:10 was selected for analysis because it was the lowest limit at which STR could detect the mixture, and matched the actual proportion of cell-free fetal DNA (cffDNA) in maternal plasma (range 5–20%, average 9–10%) [17,54]. We set 1, 3, and 5 µL of DNA input to explore the effects of sequencing genotypes corresponding to different sequencing inputs. The degraded fragment at 15 min was too short to be combined with STR genotypes, so only degradations at 2.5, 5, and 10 min were simulated. The Agilent 2100 Bioanalyzer (Agilent Technologies, Santa Clara, CA, USA) performed well in detecting degraded samples, basically conforming to the fragment distribution of STR genotypes (Supplementary Figure S4). The detection rate of minor DNA unique (effective) alleles was 93–100% in the nine simulated degraded mixtures (Table 2). When the DNA input was 1, 3, and 5 μL, the results showed that 3 and 5 μL performed better, which provided a solid theoretical basis for the DNA input in further degraded mixtures research. In addition, maternal plasma DNA containing cffNDA is a special degraded mixture essentially, in which cffNDA accounts for about 10% on average, and the median fragment length is about 143 bp owing to its apoptotic nature [55]. Therefore, we suggest that the DNA input for MPS be 3 or 5 µL to improve the detection rate for the future degraded mixtures and NIPPT study.

## 5. Conclusions

In this study, we constructed an MPS-based 50-plex MH panel for forensic DNA analysis combined with multiple PCR-targeted capture sequencing technology and a homemade calling pipeline. We comprehensively explored the potential of the panel for forensic applications, including sensitivity, accuracy, polymorphism, forensic parameters, undegraded mixtures, single-degraded samples, degraded mixtures, and species specificity. We also improved the primer optimization of our panel, explored the influence of different DNA inputs on the efficiency of MH detection in mixtures, and developed a universally applicable MHs forensic analysis software package. Furthermore, our panel characterized a new set of 49 MHs, which may contribute to an international community consensus on a possible MH core panel.

In a nutshell, the current findings demonstrated that our MPS-based 50plex MH panel is a unique and powerful DNA tool. It is also an alternative method that can complement and improve the interpretation ability of mixtures and the efficiency of kinship testing with traditional STRs. Our future studies will focus on more family sample pairs to evaluate the value of the panel in NIPPT.

## Figures and Tables

**Table 1:** Numbers of loci during the stepwise construction of the microhaplotypes (MHs) panel.

Chr	Length ≤ 80 & SNP ≥ 2	A_e_^a^ ≥ 3	A_e_ ≥ 4	MHs with the Largest A_e_ from All Overlapping Sequences in Each Group	Remove the MHs with Obvious Repeat Motifs in the Base Sequence	MHs with Physical Position ≥ 10 Mb as an Interval	MHs with Successful Primer Design	MHs Retained after Six Rounds of Optimization
1	381,886	4969	781	177	150	16	12	5
2	395,095	3944	493	139	118	17	13	5
3	332,581	3286	373	93	79	10	6	1
4	374,323	5527	1038	186	172	11	8	5
5	295,470	3066	305	98	88	10	8	3
6	411,469	19,040	3065	538	523	12	10	3
7	301,403	4717	774	173	163	8	5	1
8	289,723	4002	463	136	126	8	4	1
9	224,267	2473	231	75	67	8	5	2
10	258,278	3442	550	122	113	11	7	2
11	242,660	2826	416	94	83	6	4	1
12	230,769	2935	381	104	90	7	5	2
13	175,580	1922	183	63	55	9	7	3
14	161,185	1768	163	65	55	7	7	4
15	151,799	1823	212	45	41	5	3	1
16	182,431	3049	436	92	85	6	3	3
17	137,595	2229	489	90	80	6	4	2
18	142,572	1908	315	78	66	6	5	3
19	141,666	2361	343	87	83	3	3	1
20	104,470	1072	95	40	37	5	4	1
21	74,494	1212	175	38	36	5	5	1
22	63,641	494	60	18	18	2	0	0
Total	5,073,357	78,065	11,341	2551	2328	178	128	50

^a^: refers effective number of alleles.

**Table 2:** Summary results of MPS-MHs of two-person mixtures.

**Non-Degraded**										
**Input DNA**	**Ratio ^a^**	**Maximum Individual Alleles**	**Total Observed Alleles**	**Total Observed Alleles/Expected Alleles %**	**Maximum Individual Alleles Minor Donor**	**Observed Alleles Minor Donor**	**Observed Alleles/Maximum Alleles Minor Donor %**	**Unique Alleles Minor Donor**	**Reportable Unique Alleles Minor Donor**	**Reportable Unique Alleles Minor Donor %**
1 μL	1:1	132	132	100	84	84	100	61	61	100
	1:3	132	132	100	84	84	100	61	61	100
	1:5	132	132	100	84	84	100	61	61	100
	1:10	132	132	100	84	84	100	61	61	100
	1:20	132	132	100	84	84	100	61	61	100
	1:40	132	132	100	84	84	100	61	61	100
**Degraded**										
**Input DNA**	**Ratio ^a^-Time**	**Maximum Individual Alleles**	**Total Observed Alleles**	**Total Observed Alleles/Expected Alleles %**	**Maximum Individual Alleles Minor Donor**	**Observed Alleles Minor Donor**	**Observed Alleles/Maximum Alleles Minor Donor %**	**Unique Alleles Minor Donor**	**Reportable Unique Alleles Minor Donor**	**Reportable Unique Alleles Minor Donor %**
1 μL	1:10–2.5	132	132	100	84	84	100	61	61	100
	1:10–5	132	110	83	84	55	65	61	39	64
	1:10–10	132	128	97	84	79	94	61	57	93
	1:10–15	132	127	96	84	79	94	61	57	93
3 μL	1:10–2.5	132	132	100	84	84	100	61	61	100
	1:10–5	132	132	100	84	84	100	61	61	100
	1:10–10	132	131	99	84	83	99	61	60	98
5 μL	1:10–2.5	132	132	100	84	84	100	61	61	100
	1:10–5	132	132	100	84	84	100	61	61	100
	1:10–10	132	128	97	84	79	94	61	57	93

^a^: refers to 1 in ‘Ratio’ for 0.5 ng.

## Data Availability

The data that support the findings of this study are available from the corresponding author upon reasonable request.

## Supplementary Materials

The following supporting information can be downloaded at: https://www.mdpi.com/article/10.3390/genes14040865/s1, Table S1: Specific information on 50 MHs and 104 primers. Table S2: Summary of MHs, samples and primers of sanger sequencing. Table S3: Allele frequencies of 50 MHs based on 137 unrelated Southwest Chinese Han individuals were sequenced. Table S4: Forensic parameters of 50 MHs based on 137 unrelated Southwest Chinese Han individuals were sequenced. Table S5: The HWE results of 50 MHs based on 137 unrelated Southwest Chinese Han individuals were sequenced. Table S6: The LD values of 50 MHs based on 137 unrelated Southwest Chinese Han individuals were sequenced. Table S7: 50-MH profiles of degraded single and mixed DNAs. Table S8: Summary of MHs genotypes and DoCs for each animal sample based on the MPS platform. Figure S1: Sensitivity results. (A) Results of three replicates of 2800 M with different inputs (10, 5, 1, 0.5, 0.25, and 0.125 ng). The solid orange line on the right axis represents the overall depths of coverage (DoCs), the dashed orange line represents the average DoC, and the blue bars on the left axis represent the number of successful callings of MHs. (B) DoCs were negatively correlated with DNA inputs. Figure S2: Several other samples verifying accuracy and consistency. (A) MH-27 and sample 152; (B) MH-27 and sample C94; (C) MH-4 and sample 153; (D) MH-4 and sample C94. Each figure shows the genotypes obtained by our pipeline, Sanger sequencing, and IGV from top to bottom. The black boxes indicate the target SNPs. The screenshot only displays the physical location and length of the target MH. Figure S3: STR profiles of two-person mixtures. 1 µL of each mixture was used to obtain 1:1, 1:3, 1:5, 1:10, 1:20, and 1:40 genotypes using the AGCU EX22 Kit (from left to right). Figure S4: Degree of degradation of single and mixed DNA detected using the Agilent 2100 Bioanalyzer (Agilent Technologies, Santa Clara, CA, USA). Random individual DNA C51, C131 and its two 1:10 mixtures were treated with DNase I at 37 °C for 2.5, 5, 10, and 15 min, respectively. Then, 1 µL of each was collected to obtain the corresponding electropherogram using a High Sensitivity DNA Kit (Agilent Technologies, Santa Clara, CA, United States) (from left to right). (A) For C51, the degraded fragments were dispersed at 2.5 min, concentrated at 200 bp at 5 min, and then concentrated at about 150 bp. (B) For C131, the degraded fragments were dispersed at 2.5 min, concentrated at 150 bp at 5 min, and then concentrated in shorter fragments. (C) For 1:10–C51 + C131, the degraded fragments were dispersed at 2.5 min, concentrated at 150 bp at 5 min, and then concentrated in shorter fragments. The last picture is a summary of electropherograms. Both degraded single and mixed samples were treated with DNase I to achieve ideal simulated degradation results. Figure S5: STR profiles of degraded single and mixed DNAs. Random individual DNAs C51, C131, and its two 1:10 mixtures were treated with DNase I at 37 °C for 2.5, 5, 10 and 15 min, respectively. 1 µL of each was taken to collected to obtain the corresponding electropherogram using an AGCU EX22 kit (Applied ScienTech, Jiangsu, China) (from left to right). (A) For C51, the peak height decreased significantly at 2.5 min, dropped from 320 bp at 5 min, and then decreased gradually. (B) For C131, the peak height dropped from 280 bp at 2.5 min, from 120 bp at 5 min, and then gradually decreased. (C) For 1:10–C51 + C131, the peak height dropped from 280 bp at 2.5 min, from 150 bp at 5–10 min, and from 120 bp at 15 min. Regardless of whether degraded single or mixed samples were used, the STR kit could not obtain complete profiles at different degradation times.

## Abbreviations

ACRs	Allele coverage ratios
A_e_	Effective number of alleles
AF	Allele frequency
ALFRED	ALelle FREquency Database
CDP	Combined discrimination power
CE	Capillary electrophoresis
CHS	Chinese Southern Han
CMP	Combined match probability
CPE	Combined probability of exclusion
DoCs	Depth of coverages
DP	Discrimination power
Het	Heterozygosity
Hom	Homozygosity
HWE	Hardy–Weinberg equilibrium
IGV	Integrative Genomics Viewer
LD	Linkage disequilibrium
MAF	Minor allele frequency
MHs	Microhaplotypes
MicroHapDB	Microhaplotype Database
MP	Match probability
MPS	Massively parallel sequencing
Multi-PCR	Multiple polymerase chain reaction
NIPPT	Noninvasive prenatal paternity testing
PE	Probability of exclusion
PIC	Polymorphism information content
POI	Person of interest
SNPs	Single nucleotide polymorphisms
STRs	Short tandem repeats
TPI	Typical paternity index

## References

[1] Kidd K.K., Pakstis A.J., Speed W.C., Lagace R., Chang J., Wootton S., Ihuegbu N. (2013). Microhaplotype loci are a powerful new type of forensic marker. Forensic Sci. Int. Genet. Suppl..

[2] Kidd K.K., Pakstis A.J., Speed W.C., Lagacé R., Kidd J.R. (2014). Current sequencing technology makes microhaplotypes a powerful new type of genetic marker for forensics. Forensic Sci. Int. Genet..

[3] Qu N., Lin S., Gao Y., Liang H., Zhao H., Ou X. (2020). A microhap panel for kinship analysis through massively parallel sequencing technology. Electrophoresis.

[4] Arnheim N., Calabrese P., Nordborg M. (2003). Hot and cold spots of recombination in the human genome: The reason we should find them and how this can be achieved. Am. J. Hum. Genet..

[5] van der Gaag K.J., de Leeuw R.H., Laros J.F.J., den Dunnen J.T., de Knijff P. (2018). Short hypervariable microhaplotypes: A novel set of very short high discriminating power loci without stutter artefacts. Forensic Sci. Int. Genet..

[6] Kidd K.K., Speed W.C., Pakstis A.J., Podini D.S., Lagacé R., Chang J., Wootton S., Haigh E., Soundararajan U. (2017). Evaluating 130 microhaplotypes across a global set of 83 populations. Forensic Sci. Int. Genet..

[7] Oldoni F., Kidd K.K., Podini D. (2019). Microhaplotypes in forensic genetics. Forensic Sci. Int. Genet..

[8] Bennett L., Oldoni F., Long K., Cisana S., Madella K., Wootton S., Chang J., Hasegawa R., Lagacé R., Kidd K.K. (2019). Mixture deconvolution by massively parallel sequencing of microhaplotypes. Int. J. Leg. Med..

[9] Cheung E.Y.Y., Phillips C., Eduardoff M., Lareu M.V., McNevin D. (2019). Performance of ancestry-informative SNP and microhaplotype markers. Forensic Sci. Int. Genet..

[10] Zhu J., Chen P., Qu S., Wang Y., Jian H., Cao S., Liu Y., Zhang R., Lv M., Liang W. (2019). Evaluation of the microhaplotype markers in kinship analysis. Electrophoresis.

[11] Zhu J., Lv M., Zhou N., Chen D., Jiang Y., Wang L., He W., Peng D., Li Z., Qu S. (2019). Genotyping polymorphic microhaplotype markers through the Illumina (®) MiSeq platform for forensics. Forensic Sci. Int. Genet..

[12] Tan M., Xue J., Zhrang R., Jian H., Xiao Y., Liu G., Zheng Y., Wu Q., Qu S., Liang W. (2022). An NGS-based microhaplotype system with high polymorphism for forensic DNA mixtures analysis. Forensic Sci. Int. Genet. Suppl. Ser..

[13] Butler J.M. (2010). Overview and History of DNA Typing. Fundamentals of Forensic DNA Typing.

[14] Oldoni F., Bader D., Fantinato C., Wootton S.C., Lagacé R., Kidd K.K., Podini D. (2020). A sequence-based 74plex microhaplotype assay for analysis of forensic DNA mixtures. Forensic Sci. Int. Genet..

[15] Stephens M., Smith N.J., Donnelly P. (2001). A new statistical method for haplotype reconstruction from population data. Am. J. Hum. Genet..

[16] Zhang R., Tan Y., Jian H., Qu S., Liu Y., Zhu J., Wang L., Lv M., Liao M., Zhang L. (2020). A new approach to detect a set of SNP-SNP markers: Combining ARMS-PCR with SNaPshot technology. Electrophoresis.

[17] Zhang R., Tan Y., Wang L., Jian H., Zhu J., Xiao Y., Tan M., Xue J., Yang F., Liang W. (2022). Set of 15 SNP-SNP Markers for Detection of Unbalanced Degraded DNA Mixtures and Noninvasive Prenatal Paternity Testing. Front. Genet..

[18] Kidd K.K., Speed W.C. (2015). Criteria for selecting microhaplotypes: Mixture detection and deconvolution. Investig. Genet..

[19] Chen P., Deng C., Li Z., Pu Y., Yang J., Yu Y., Li K., Li D., Liang W., Zhang L. (2019). A microhaplotypes panel for massively parallel sequencing analysis of DNA mixtures. Forensic Sci. Int. Genet..

[20] Pang J.B., Rao M., Chen Q.F., Ji A.Q., Zhang C., Kang K.L., Wu H., Ye J., Nie S.J., Wang L. (2020). A 124-plex Microhaplotype Panel Based on Next-generation Sequencing Developed for Forensic Applications. Sci. Rep..

[21] de la Puente M., Phillips C., Xavier C., Amigo J., Carracedo A., Parson W., Lareu M.V. (2020). Building a custom large-scale panel of novel microhaplotypes for forensic identification using MiSeq and Ion S5 massively parallel sequencing systems. Forensic Sci. Int. Genet..

[22] Turchi C., Melchionda F., Pesaresi M., Tagliabracci A. (2019). Evaluation of a microhaplotypes panel for forensic genetics using massive parallel sequencing technology. Forensic Sci. Int. Genet..

[23] Gandotra N., Speed W.C., Qin W., Tang Y., Pakstis A.J., Kidd K.K., Scharfe C. (2020). Validation of novel forensic DNA markers using multiplex microhaplotype sequencing. Forensic Sci. Int. Genet..

[24] Butler J.M. (2014). Advanced Topics in Forensic DNA Typing: Interpretation.

[25] Osier M.V., Cheung K.H., Kidd J.R., Pakstis A.J., Miller P.L., Kidd K.K. (2001). ALFRED: An allele frequency database for diverse populations and DNA polymorphisms—An update. Nucleic Acids Res..

[26] Standage D.S., Mitchell R.N. (2020). MicroHapDB: A Portable and Extensible Database of All Published Microhaplotype Marker and Frequency Data. Front. Genet..

[27] Xue J., Qu S., Tan M., Xiao Y., Zhang R., Chen D., Lv M., Zhang Y., Zhang L., Liang W. (2022). An overview of SNP-SNP microhaplotypes in the 26 populations of the 1000 Genomes Project. Int. J. Leg. Med..

[28] Zhang B., Li Z., Li K., Chen P., Chen F. (2019). Forensic parameters and mutation analysis of 23 short tandem repeat (PowerPlex® Fusion System) loci in Fujian Han Chinese population. Leg. Med..

[29] Gao Z., Chen X., Zhao Y., Zhao X., Zhang S., Yang Y., Wang Y., Zhang J. (2018). Forensic genetic informativeness of an SNP panel consisting of 19 multi-allelic SNPs. Forensic Sci. Int. Genet..

[30] Sun S., Liu Y., Li J., Yang Z., Wen D., Liang W., Yan Y., Yu H., Cai J., Zha L. (2020). Development and application of a nonbinary SNP-based microhaplotype panel for paternity testing involving close relatives. Forensic Sci. Int. Genet..

[31] Tao R., Yang Q., Xia R., Zhang X., Chen A., Li C., Zhang S. (2022). A sequence-based 163plex microhaplotype assay for forensic DNA analysis. Front. Genet..

[32] Qu S., Lv M., Xue J., Zhu J., Wang L., Jian H., Liu Y., Zhang R., Zha L., Liang W. (2020). Multi-Indel: A Microhaplotype Marker Can Be Typed Using Capillary Electrophoresis Platforms. Front. Genet..

[33] Wang K., Li H., Xu Y., Shao Q., Yi J., Wang R., Cai W., Hang X., Zhang C., Cai H. (2019). MFEprimer-3.0: Quality control for PCR primers. Nucleic Acids Res..

[34] Li H., Durbin R. (2009). Fast and accurate short read alignment with Burrows-Wheeler transform. Bioinformatics.

[35] Li H., Handsaker B., Wysoker A., Fennell T., Ruan J., Homer N., Marth G., Abecasis G., Durbin R. (2009). The Sequence Alignment/Map format and SAMtools. Bioinformatics.

[36] Zhao F., Wu X., Cai G., Xu C. (2003). The application of Modified-Powerstates software in forensic biostatistics. Chin. J. Forensic Med..

[37] Excoffier L., Lischer H.E. (2010). Arlequin suite ver 3.5: A new series of programs to perform population genetics analyses under Linux and Windows. Mol. Ecol. Resour..

[38] Duke K.R., Myers S.P. (2020). Systematic evaluation of STRmix™ performance on degraded DNA profile data. Forensic Sci. Int. Genet..

[39] Kidd K.K., Pakstis A.J., Speed W.C., Lagace R., Wootton S., Chang J. (2018). Selecting microhaplotypes optimized for different purposes. Electrophoresis.

[40] Jian H., Wang L., Lv M., Tan Y., Zhang R., Qu S., Wang J., Zha L., Zhang L., Liang W. (2021). A Novel SNP-STR System Based on a Capillary Electrophoresis Platform. Front. Genet..

[41] Zhang C., Cao Y.-D., Song J.-J., Rao M., Nie S.-J., Zhang G.-F., Kang K.-L., Ji A.-Q., Ye J., Wang L. (2019). MHTyper: A microhaplotype allele-calling pipeline for use with next generation sequencing data. Aust. J. Forensic Sci..

[42] Ou X., Qu N. (2020). Noninvasive prenatal paternity testing by target sequencing microhaps. Forensic Sci. Int. Genet..

[43] Kwon Y.L., Lee E.Y., Kim B.M., Joo S.M., Jeong K.S., Chun B.W., Lee Y.H., Park K.W., Shin K.J. (2022). Application of a custom haplotype caller to analyze sequence-based data of 56 microhaplotypes. Forensic Sci. Int. Genet..

[44] Yang J., Chen J., Ji Q., Yu Y., Li K., Kong X., Xie S., Zhan W., Mao Z., Yu Y. (2022). A highly polymorphic panel of 40-plex microhaplotypes for the Chinese Han population and its application in estimating the number of contributors in DNA mixtures. Forensic Sci. Int. Genet..

[45] Jin X.Y., Cui W., Chen C., Guo Y.X., Zhang X.R., Xing G.H., Lan J.W., Zhu B.F. (2020). Developing and population analysis of a new multiplex panel of 18 microhaplotypes and compound markers using next generation sequencing and its application in the Shaanxi Han population. Electrophoresis.

[46] Zou X., He G., Liu J., Jiang L., Wang M., Chen P., Hou Y., Wang Z. (2022). Screening and selection of 21 novel microhaplotype markers for ancestry inference in ten Chinese subpopulations. Forensic Sci. Int. Genet..

[47] Oldoni F., Yoon L., Wootton S.C., Lagacé R., Kidd K.K., Podini D. (2020). Population genetic data of 74 microhaplotypes in four major U.S. population groups. Forensic Sci. Int. Genet..

[48] Alonso A., Barrio P.A., Müller P., Köcher S., Berger B., Martin P., Bodner M., Willuweit S., Parson W., Roewer L. (2018). Current state-of-art of STR sequencing in forensic genetics. Electrophoresis.

[49] Kidd K.K., Pakstis A.J. (2022). State of the Art for Microhaplotypes. Genes.

[50] Neyra-Rivera C.D., Ticona Arenas A., Delgado Ramos E., Velasquez Reinoso M.R.E., Budowle B. (2021). Allelic frequencies with 23 autosomic STRS in the Aymara population of Peru. Int. J. Leg. Med..

[51] Srivastava A., Nath S., Das K.K., Kumar A., Kushwaha P., Kumar A., Srivastav K.V.V., Bhasney V., Rana M., Dixit S. (2021). Forensic characterization and genomic diversity of Assam population viewed from 23 autosomal STRs. Int. J. Leg. Med..

[52] Bae S., Won S., Kim H. (2021). Selection and evaluation of bi-allelic autosomal SNP markers for paternity testing in Koreans. Int. J. Leg. Med..

[53] Wen D., Xing H., Liu Y., Li J., Qu W., He W., Wang C., Xu R., Liu Y., Jia H. (2022). The application of short and highly polymorphic microhaplotype loci in paternity testing and sibling testing of temperature-dependent degraded samples. Front. Genet..

[54] Tan Y., Zhang L., Bai P., Li Z., Zhang R., Yang F., Wang L., Liang W. (2021). Detection of cell-free fetal DNA in maternal plasma using two types of compound markers. Electrophoresis.

[55] Lo Y.M., Chan K.C., Sun H., Chen E.Z., Jiang P., Lun F.M., Zheng Y.W., Leung T.Y., Lau T.K., Cantor C.R. (2010). Maternal plasma DNA sequencing reveals the genome-wide genetic and mutational profile of the fetus. Sci. Transl. Med..

