# Supplementary material for: An MPS-Based 50plex Microhaplotype Assay for Forensic DNA Analysis
Source: Genes (Basel). 2023 Apr 4;14(4):865. doi: 10.3390/genes14040865 (PMC10137789; doi:10.3390/genes14040865)
Supplement: Supplementary file 1 [file genes-14-00865-s001.zip › Supplementary Figures.pdf]

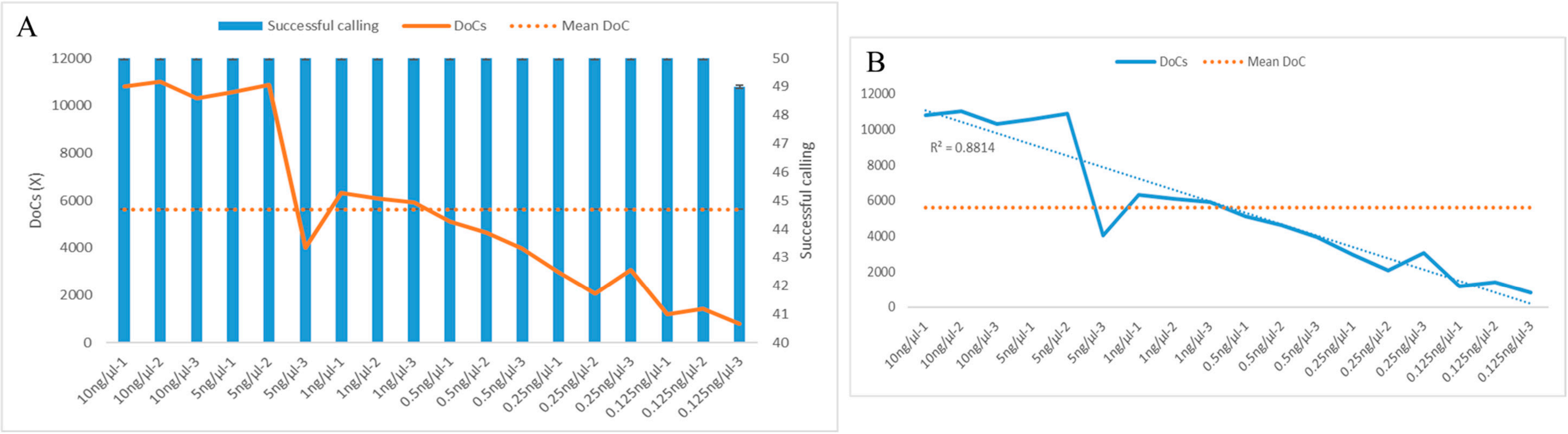

Supplementary Figure S1. Sensitivity results. (**A**) Results of three replicates of 2800M with different inputs (10, 5, 1, 0.5, 0.25, and 0.125 ng). The solid orange line on the right axis represents the overall depth of coverages (DoCs), the dashed orange line represents the average DoC, and the blue bars on the left axis represent the number of successful calling of microhaplotypes (MHs). (**B**) DoCs were negatively correlated with DNA inputs.

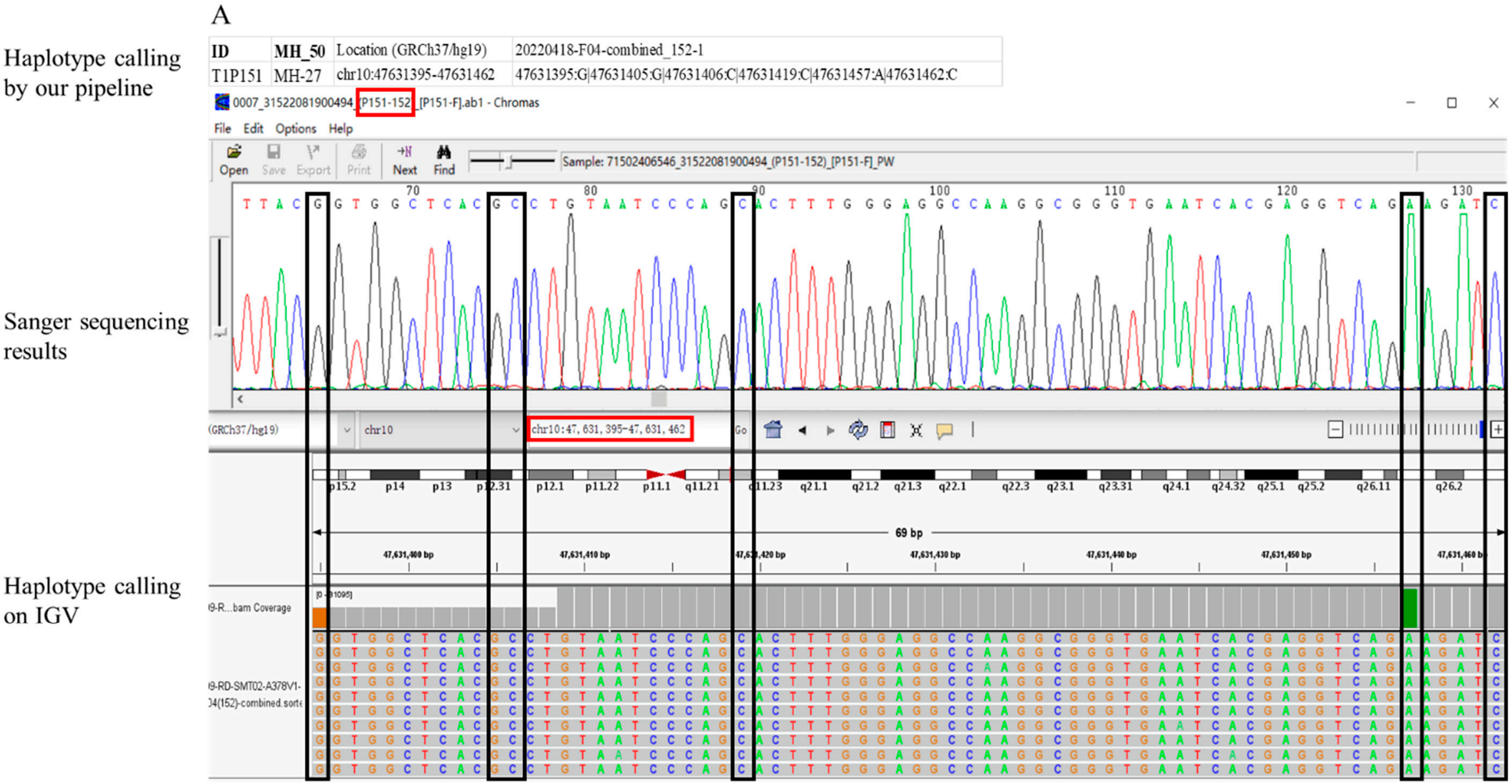
A
Haplotype calling by our pipeline
ID | MH_50 | Location (GRCh37/hg19) | 20220418-F04-combined_152-1
T1P151 | MH-27 | chr10:47631395-47631462 | 47631395:G|47631405:G|47631406:C|47631419:C|47631457:A|47631462:C
0007_31522081900494_[P151-152]_[P151-F].ab1 - Chromas
File Edit Options Help
Open Save Export Print Next Find
Sample: 71502406546_31522081900494_(P151-152)_[P151-F]_PW
Sanger sequencing results
(GRCh37/hg19)
chr10
chr10:47,631,395-47,631,462
Go
69 bp
Haplotype calling on IGV

B

Haplotype calling by our pipeline

ID	MH_50	Location (GRCh37/hg19)	0A-PM1-22R28383_C94
T1P151	MH-27	chr10:47631395-47631462	47631395:A|47631405:G|47631406:C|47631419:C|47631457:G|47631462:C<br>47631395:G|47631405:G|47631406:C|47631419:C|47631457:G|47631462:C

Sanger sequencing results

Haplotype calling on IGV

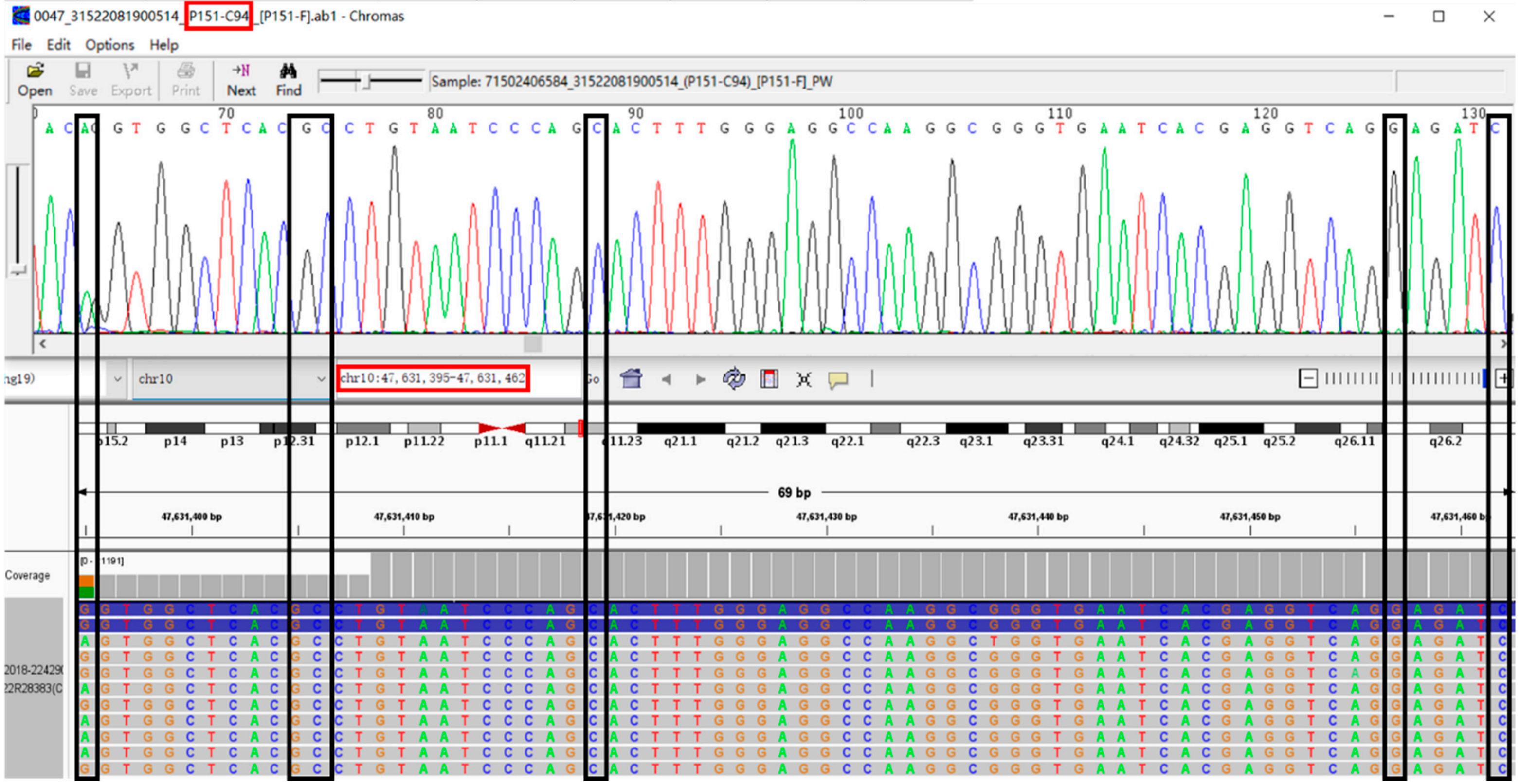

C

Haplotype calling by our pipeline

<table>
<tr><td>ID</td><td>MH_50</td><td>Location (GRCh37/hg19)</td><td>20220418-F05-combined_153-1</td></tr>
<tr><td>T1P14</td><td>MH-4</td><td>chr1:218265683-218265718</td><td>218265683:C|218265713:G|218265718:C
218265683:C|218265713:A|218265718:C</td></tr>
</table>

Sanger sequencing results

Haplotype calling on IGV

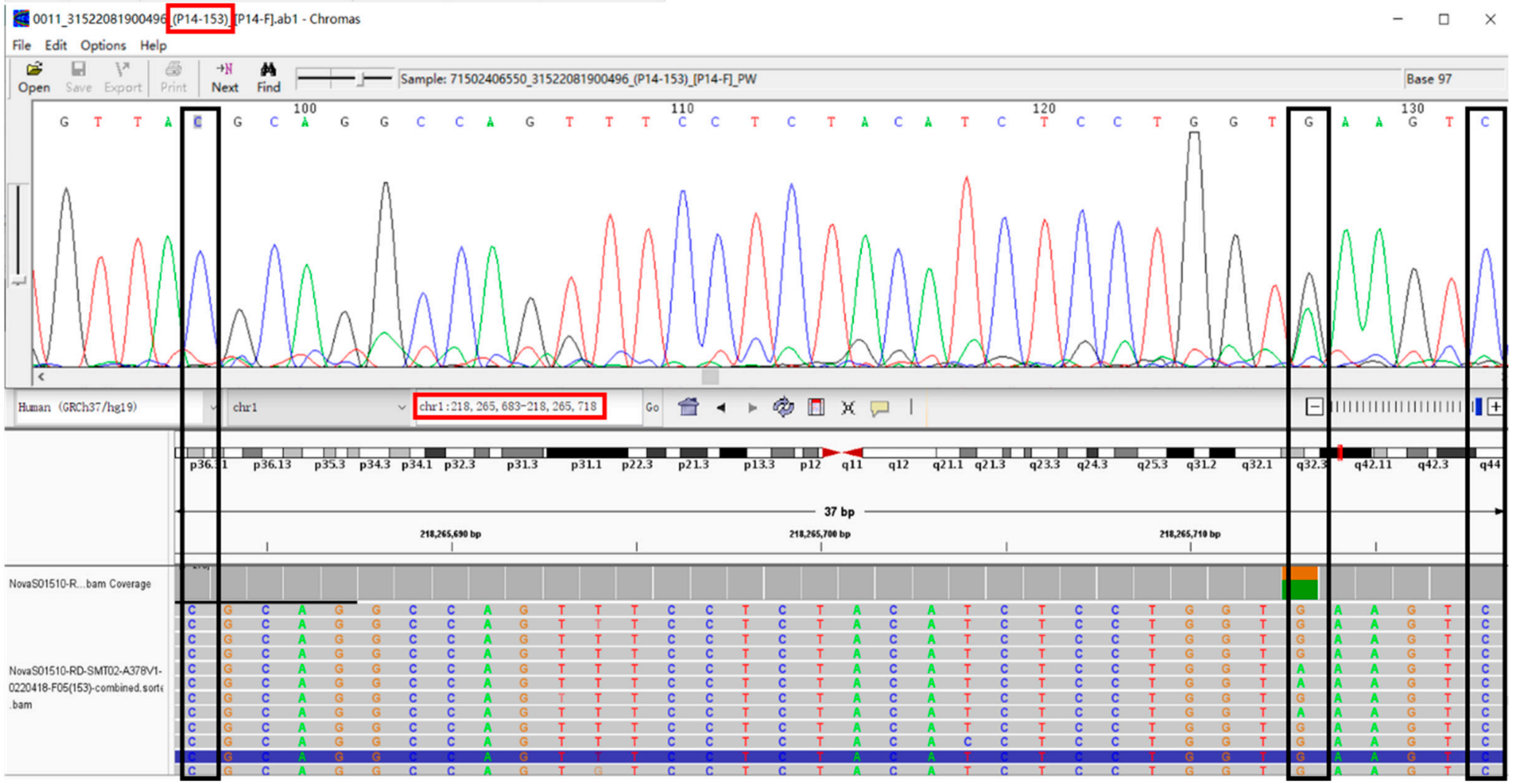

D

Haplotype calling by our pipeline

<table>
<tr><th>ID</th><th>MH_50</th><th>Location (GRCh37/hg19)</th><th>0A-PM1-22R28383_C94</th></tr>
<tr><td>T1P14</td><td>MH-4</td><td>chr1:218265683-218265718</td><td>218265683:C|218265713:A|218265718:C
218265683:C|218265713:G|218265718:A</td></tr>
</table>

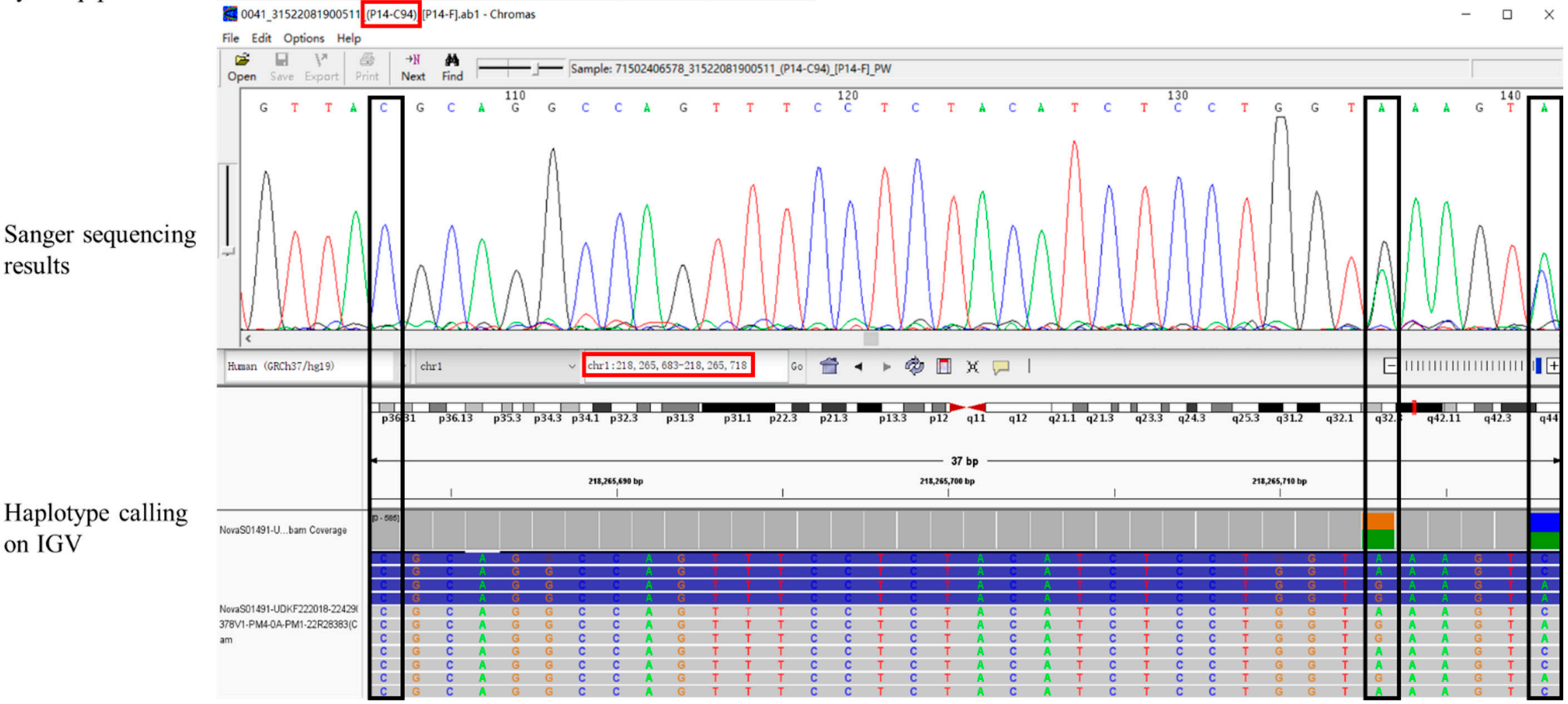

Supplementary Figure S2. Several other samples verifying accuracy and consistency. (**A**) MH-27 and sample 152; (**B**) MH-27 and sample C94; (**C**) MH-4 and sample 153; (**D**) MH-4 and sample C94. Each figure shows the genotypes obtained by our pipeline, Sanger sequencing, and Integrative Genomics Viewer (IGV) from top to bottom. The black boxes indicate the target SNPs. The red boxes represent "MH ID-Sample ID" and "Location (GRCh37/hg19)",

respectively. The screenshots only display the physical location and length of the target MH.

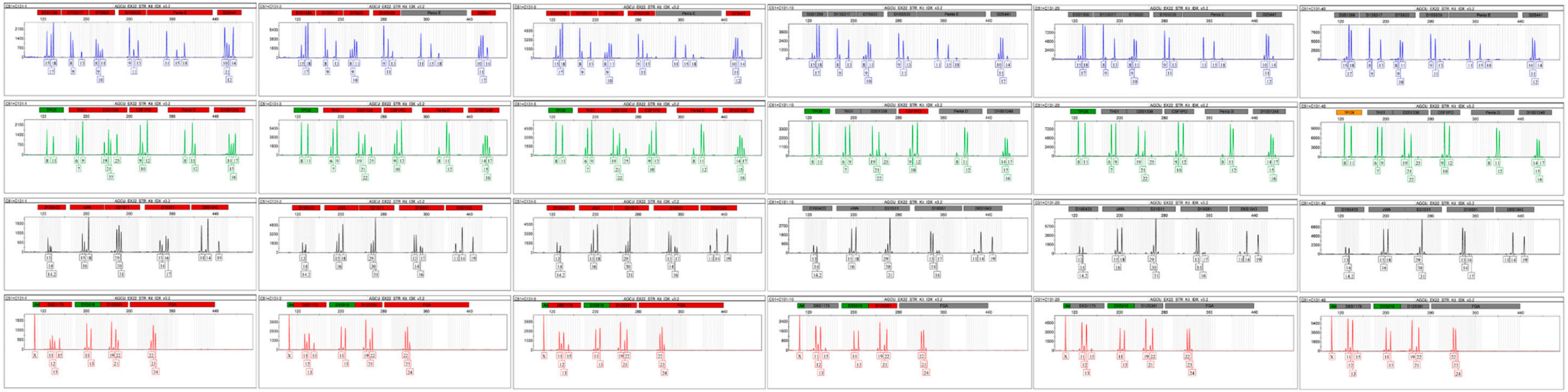

Supplementary Figure S3. STR profiles of two-person mixtures. 1 μL of each mixture was used to obtain 1:1, 1:3, 1:5, 1:10, 1:20, and 1:40 genotypes from left to right using the AGCU EX22 Kit (Applied ScienTech, Suzhou, Jiangsu, China).

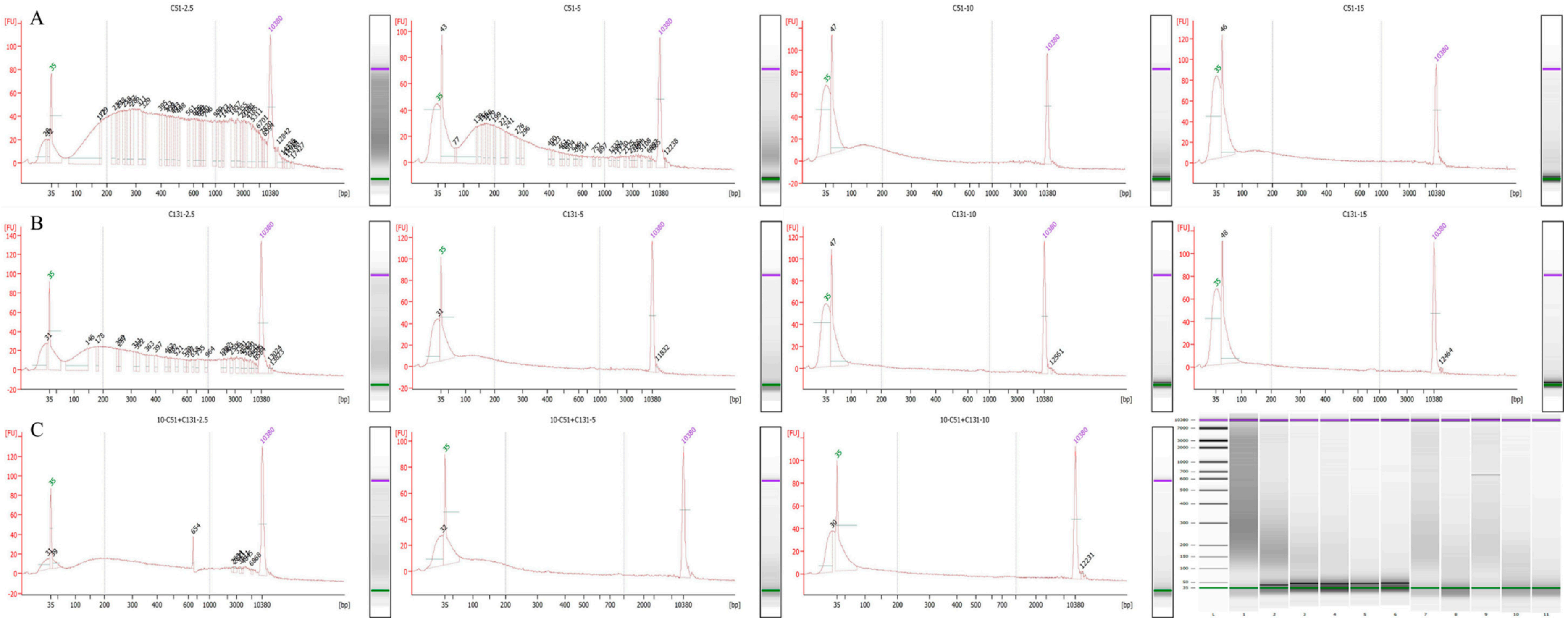

Supplementary Figure S4. Degree of degradation of single and mixed DNA detected using the Agilent 2100 Bioanalyzer (Agilent Technologies, Santa Clara, CA, USA). Random individual DNA C51, C131 and its two 1:10 mixtures were treated with DNase I at 37 ℃ for 2.5, 5, 10, and 15 min, respectively. Then, 1 μL of each was collected to obtain the corresponding electropherogram from left to right using a High Sensitivity DNA Kit (Agilent Technologies, Santa Clara, CA, USA). (**A**) For C51, the degraded fragments were dispersed at 2.5 min, concentrated at 200 bp at 5 min, and then concentrated at about 150 bp. (**B**) For C131, the degraded fragments were dispersed at 2.5 min, concentrated at 150 bp at 5 min, and then concentrated in shorter fragments. (**C**) For 1:10–C51+C131, the degraded fragments were dispersed at 2.5 min, concentrated at 150 bp at 5 min, and then concentrated in shorter fragments. The last picture is a summary of electropherograms. Both degraded single and mixed samples were treated with DNase I to achieve ideal simulated degradation results.

A

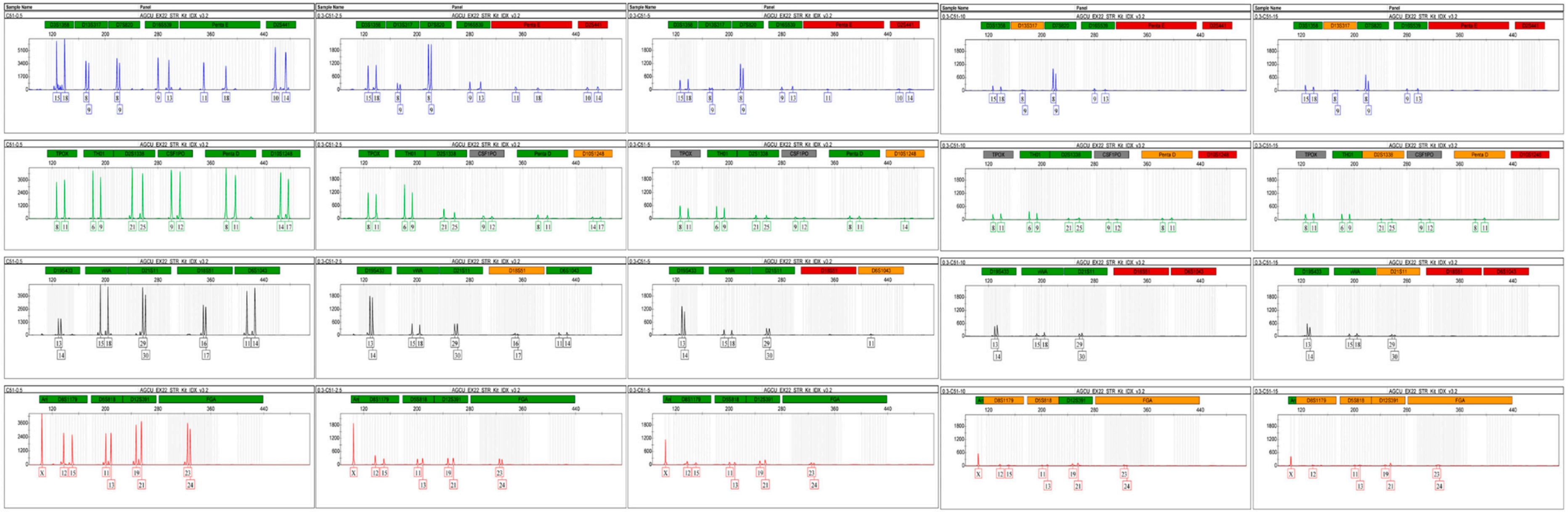

B

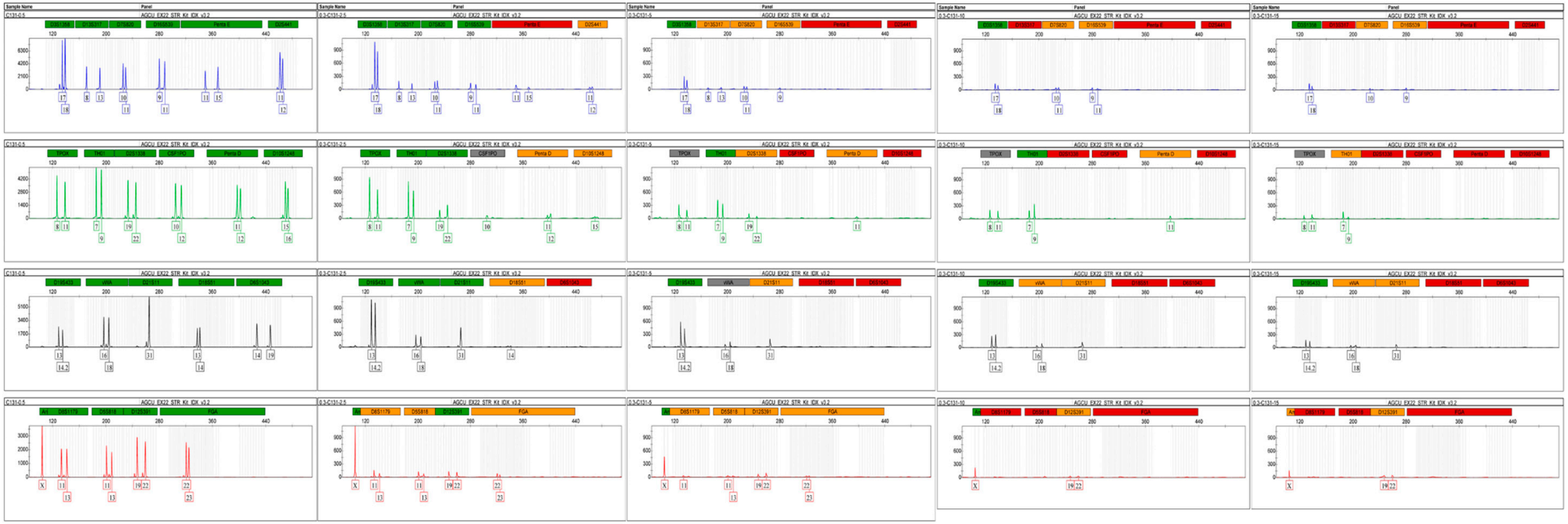

C

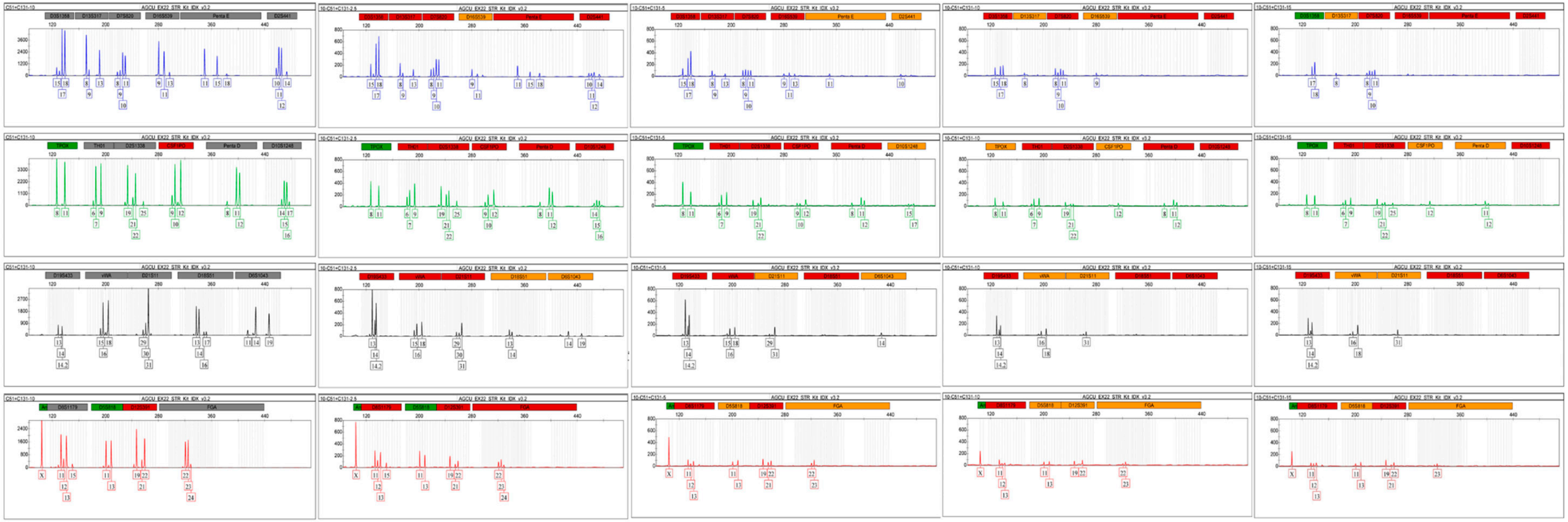

Supplementary Figure S5. STR profiles of degraded single and mixed DNAs. Random individual DNAs C51, C131, and its two 1:10 mixtures were treated with DNase I at 37 ℃ for 2.5, 5, 10 and 15 min, respectively. 1 μL of each was taken to collected to obtain the corresponding electropherogram using an AGCU EX22 kit (from left to right). (**A**) For C51, the peak height decreased significantly at 2.5 min, dropped from 320 bp at 5 min, and then decreased gradually. (**B**) For C131, the peak height dropped from 280 bp at 2.5 min, from 120 bp at 5 min, and then gradually decreased. (**C**) For 1:10–C51+C131, the peak height dropped from 280 bp at 2.5 min, from 150 bp at 5–10 min, and from 120 bp at 15 min. Regardless of whether degraded single or mixed samples were used, the STR kit could not obtain complete profiles at different degradation times.
